# Human Papillomavirus Induced Transformation in Cervical and Head and Neck Cancers

**DOI:** 10.3390/cancers6031793

**Published:** 2014-09-15

**Authors:** Allie K. Adams, Trisha M. Wise-Draper, Susanne I. Wells

**Affiliations:** 1 Cancer and Blood Diseases Institute, Cincinnati Children’s Hospital Medical Center, Cincinnati, OH 45229, USA; E-Mail: allie.varner@cchmc.org; 2 Division of Hematology/Oncology, University of Cincinnati Medical Center, University of Cincinnati, Cincinnati, OH 45229, USA; E-Mail: wiseth@uc.edu

**Keywords:** human papillomavirus, cervical cancer, head and neck cancer

## Abstract

Human papillomavirus (HPV) is one of the most widely publicized and researched pathogenic DNA viruses. For decades, HPV research has focused on transforming viral activities in cervical cancer. During the past 15 years, however, HPV has also emerged as a major etiological agent in cancers of the head and neck, in particular squamous cell carcinoma. Even with significant strides achieved towards the screening and treatment of cervical cancer, and preventive vaccines, cervical cancer remains the leading cause of cancer-associated deaths for women in developing countries. Furthermore, routine screens are not available for those at risk of head and neck cancer. The current expectation is that HPV vaccination will prevent not only cervical, but also head and neck cancers. In order to determine if previous cervical cancer models for HPV infection and transformation are directly applicable to head and neck cancer, clinical and molecular disease aspects must be carefully compared. In this review, we briefly discuss the cervical and head and neck cancer literature to highlight clinical and genomic commonalities. Differences in prognosis, staging and treatment, as well as comparisons of mutational profiles, viral integration patterns, and alterations in gene expression will be addressed.

## 1. Introduction

Human papillomaviruses (HPVs) comprise a large family of viruses, 170 of which are now sequenced and are extensively referenced on the Papillomavirus Episteme [1,2]. The high risk (HR) mucosal HPV types, including the HPV16 prototype, are a subgroup of the alpha papillomaviruses that share the ability to cause cancer in their human host [3]. These DNA viruses have been extensively investigated for decades for their ability to subvert cellular mechanisms of growth control. In fact, HPV was first implicated in cancer biology approximately forty years ago by Harald zur Hausen, who was awarded the Nobel Prize in Physiology or Medicine in 2008 for the discovery that HPV causes cervical cancer [4,5]. Since then, HPV has also been associated with other anogenital cancers including vulvar, penile, and anal, largely due to sexual transmission. More recently, high risk HPV has been clearly detected in head and neck cancers (HNCs), particularly in the oropharynx, making it an additional etiological factor for a disease previously ascribed most commonly to tobacco and alcohol consumption. Both cervical and head and neck malignancies have poor outcomes when diagnosed at late stages and there is a dire need for clinically effective screening methods for detection of early stage disease and for the identification of novel therapeutic targets.

Cervical cancer in the United States has been on a swift decline due to the general implementation of Papanicolaou (Pap) screening for the detection of pre-malignant lesions of the cervix. However, the expense of treating HPV-related infections is a significant burden to society. Approximately $8 billion was spent for the year 2010, with the majority focused on screening and follow-up visits, and with just over 10% of the total expense on the treatment of cervical cancer [6]. Many lives are saved in return on a national level, but cervical cancer remains a major cause of death in developing countries where vaccination and screening programs are unavailable. Furthermore, similar screens are not currently available to detect early HPV positive (or negative) head and neck cancers. Clinicians must rely on surrogate markers, such as the cyclin-dependent kinase inhibitor p16 (INK4a), to determine HPV status in the tumor. HPV negative (HPV^−^) HNCs have been steadily declining in correlation with decreased tobacco use, while HPV positive (HPV^+^) head and neck cancers are on a steep, upward trajectory especially in younger populations, and are estimated to surpass cervical cancer rates in the U.S. by the year 2020 [7]. The increasing incidence of HPV^+^ HNCs is linked to increasing oral HPV exposure via oral sex in younger age groups, and the total number of oral sex and open-mouthed (“French”) kissing partners [8]. As for cervical cancer, the risk of HPV infection also increases with greater numbers of sexual partners. Given the prospect of a national epidemic of HPV^+^ HNCs, it is now critical to test the possibility that protection from cervical cancer through HPV vaccination will also be protective against HNC. For many years, the study of HPV in cancer biology has been focused on cervical cancers. With a large upward shift in the number of HPV positive HNCs it will be imperative to adapt the paradigms of HPV function in cervical cancer to HNC wherever possible.

Almost all cervical and at least one-quarter of head and neck cancers share the presence of HPV genomic DNA and expression of the viral oncogenes. A majority of both cervical and HPV^+^ HNCs are squamous cell carcinomas (SCCs) due to keratinocyte-specific viral tropism. Advances in the prevention and treatment of cervical cancer may be an excellent starting point for battling HPV positive HNC, but biological distinctions between these cancer types exist and must be clearly defined in order to apply this knowledge more broadly. For example, HPV types are much more restricted in head and neck cancer when compared to cervical cancer, mostly due to HPV-16 infection varying in its distribution (*i.e*., prevalence) predominantly as a function of HNC site (e.g., tonsillar *versus* oral cavity) [9,10,11]. Furthermore, cervical cancers have shown a dependence on estrogen signaling, whereas hormonal contributions have not been identified in HNC [12,13,14,15,16]. Here, we discuss the clinical and molecular commonalities and differences between HPV^+^ cervical and head and neck squamous cell carcinomas (HNSCCs).

## 2. HPV Biology

Human papillomavirus is an episomal, double-stranded DNA virus. Almost 200 known types have been identified, only some of which contribute to malignancies. The majority of HPV types infect the non-mucosal, cutaneous epithelium [3]. The minority of HPV types that infect mucosal tissues and the genital tract are sub-divided into high risk and low risk categories based on their ability to transform the host cell. HPV16, 18, 31, and 45 are examples of high-risk types and are known to cause cervical neoplasias and tumors, while HPV6 and 11 belong to the low-risk subtypes that induce genital warts and respiratory papillomas [17]. There are also HPV16 variants that are classified based on sequence alterations in E6 and are detected in different regions across the globe and are named correspondingly. These include: European (EUR), Asian American (AA), Asian (As), African-1 (AFR1), and African-2 (AFR2) variants [18,19,20]. These variants appear to exhibit differences in their relative contributions to cervical malignancies, with Asian American variants preferentially associated with high-grade cervical intraepithelial neoplasia (CIN) and cervical cancer [21,22,23,24,25]. Research in HNSCC on HPV16 variants has been more limited, although recent studies implicate certain variants, such as R10G, to be more frequent in HNSCC than in cervical cancer [26,27]. New variants have been identified in HNSCC, but most are shared with those already identified [9].

The HPV genome is circular with dual promoters that encode two separate groups of viral proteins: the early genes (E1, E2, E4, E5, E6, E7, E8) and the late genes (L1, L2). Some of the early HPV genes are essential for maintaining the viral replicative cycle, while the late HPV genes encode the major (L1) and minor (L2) capsid proteins [28]. E1 and E2 are primarily involved in transcription and replication: E1 functions as the viral DNA helicase and E2 as a transcriptional activator and repressor that also complexes with E1 as a critical component of the HPV replisome [29,30,31,32]. The transforming properties of HPV are a result of E5, E6, and E7 activities, which are discussed in further detail below. The HPV E4 protein is less well characterized, but several studies implicate E4 in virion release via its association with keratin filaments [33,34].

The epithelium is comprised of proliferating and differentiated keratinocytes, which are the host cells for HPV infection and replication, respectively. Keratinocytes that transition into malignant cells via immortalization and transformation initiated by the presence of high-risk HPV frequently result in squamous cell carcinoma (SCC). Transmission of HPV relies on micro-wounds or abrasions in the epidermis for HPV to gain access to stem and/or proliferating cells in the basal epithelium. Once there, it will utilize the host cell replication machinery to initiate viral DNA replication [35]. Our understanding of the initial HPV infection process including viral attachment and entry remains incomplete, but interactions with heparan sulfate on the cell surface have been implicated [36,37,38,39,40,41,42]. The HPV life cycle is highly organized, geographically as well as functionally, around the cellular differentiation program [43,44]. Under normal circumstances, cell division in the basal cell layer regenerates the stem and transit amplifying cell population, with a subset of cells separating to move upwards, exit the cell cycle and differentiate, thus forming the spinous, granular and cornified layers of the epidermis. In the HPV infected environment, expression of early HPV genes, particularly E7, forces re-entry into the cell cycle, thus enabling HPV to take advantage of active host replication machinery to amplify its viral genome [45,46]. One mechanism by which HPV regulates viral genome copy number is through an E2 variant, E8^E2C. E8^E2C is a transcriptional repressor identified in high-risk HPV types 31 and 16 that limits viral genome replication, and is required for HPV31, but not HPV16 [47,48,49]. Once HPV-infected cells reach the upper layers of the epithelium, late viral genes are expressed for capsid production and virion assembly, and release of infectious particles follows. HPV is shed from the surface of the epidermis in conjunction with squamous flakes in order to maximize local virus concentrations. As such, new infections can occur to repeat the infectious cycle in the same or in a different host.

Over the course of viral replication, HPV is maintained as an extrachromosomal, circular element (episome). Partitioning to daughter cells during mitosis can be attributed, at least in part, to the function of E2. The E2 protein is able to tether the HPV genome to DNA through the binding of cellular DNA-associated factors including, but not limited to, Brd4 [50,51]. Episomal HPV is often detected in non-malignant and pre-malignant tissues, while integrated HPV is detected largely in malignancies, but is not necessarily required for oncogenic progression. This is highlighted by one study examining the frequency of HPV integration in precancerous and cancerous cervical lesions. The three most common high-risk HPV types (16, 18, and 45) were integrated more often relative to other high-risk HPV types. Furthermore, HPV45 was most frequently integrated in precancerous lesions, while HPV18 was most frequently integrated in cervical cancer. Although HPV was present in all specimens, integration was not ubiquitously detected suggesting integration-independent mechanisms of HPV-induced oncogenesis are at play [52]. HPV has been reported to integrate in the host DNA at common fragile sites, although the copy number and location varies [53]. However, other mechanisms of HPV integration have been proposed recently, based on sophisticated genome sequencing technology, in cervical and head and neck cancers. These will be described in further detail below.

Integration events can disrupt E2, allowing for the deregulation of the HPV E6 and E7 oncogenes whose upregulation then further promotes growth advantages and genomic instability through their respective cellular targets [54,55]. E2 controls E6 and E7 expression in part through promoter binding and repression [56,57,58,59]. Once released from E2-mediated repression, E6 and E7 activities are stimulated. These proteins bind and target p53 and Rb pocket proteins for degradation, respectively. E7 not only binds to Rb, but also to the Rb family members p107 and p130, ensuring cells progress through S-phase in the absence of proper E2F/Rb cell-cycle control, and gain proliferative characteristics [60,61]. E6 also activates telomerase reverse transcriptase (TERT), which contributes to keratinocyte immortalization [62,63,64]. More recent studies of these oncogenes have identified new targets in various signaling pathways. For instance, the importance of hormone signaling in the development of cervical cancers is clearly appreciated. A requirement for the estrogen receptor (ER) was discovered in transgenic K14E7 mouse models for the initiation and maintenance of cervical cancer [13,16]. Furthermore, microRNAs (miRNAs) are emerging as an additional mechanism whereby the HPV oncogenes promote SCC [65,66,67,68]. More detailed information on the numerous targets of E6 and E7 can be found in elegant reviews on the biology of HPV [46,69,70,71].

Expression of the HPV E5 oncogene is also believed to contribute to the oncogenicity of HPV, at least in part through signaling from growth factor receptors such as EGFR [72]. *In vivo* experiments suggest E5 contributes to hyperplasia, aids in the formation of epithelial tumors and synergizes with E6 and E7 to promote more severe disease phenotypes [73,74,75]. Recent work also demonstrated E5 regulates growth and invasion in cervical cancer cell lines [76]. The interplay between HPV E5, E6 and E7, and their downstream targets is critical for enabling HPV to immortalize and transform keratinocytes, eventually leading to SCC formation.

While there is ample data on HPV viral activities and cellular targets in the literature, details on the viral life cycle including mechanisms of uptake, replication and virion production remain to be discovered. Additionally, evolutionarily conserved properties and critical molecular distinctions between high risk and low risk HPVs require further study for an improved understanding of disease etiology [77,78]. The capacity of low-risk HPV E6 and E7 proteins to bind and degrade p53 and Rb is limited when compared to that of the corresponding high-risk HPV proteins. Low risk HPV E6 proteins also do not harbor PDZ binding domains, which contribute to the transforming ability of E6 [79,80]. Furthermore, the characteristics of basal cells that are initially infected by HPV have remained controversial. The basal compartment of the epidermis is believed to contain bona fide stem cells with low proliferative capacity and highly proliferative transit amplifying (TA) cells with less of a self-renewal capacity. The existence of TA cells has been debated, but it is believed these cells will only proliferate for a limited time and eventually undergo differentiation [81,82]. It remains to be seen whether and how HPV targets the stem TA cell population in the human epidermis [44,83,84,85]. Finally, HPV has been detected in multiple cancer types including cervical, anogenital and head and neck, but the extent to which tissue specific molecular activities drive cancer phenotypes remains unclear.

## 3. Clinical Characteristics of HPV^+^ Cancers

### 3.1. Etiology of Cervical and Head and Neck Cancers

#### 3.1.1. Cervical Cancer

Cervical cancer is the second leading cause of female cancer deaths worldwide, but due to national recommendations for screening and vaccination the burden of cervical cancer lies largely outside of the United States. HPV accounts for nearly 100% of all cervical cancers, most of which are squamous cell carcinomas due to infection with high risk HPV16; however, cervical adenocarcinomas are most strongly correlated with infection of high risk HPV18 (Table 1) [86,87]. Although the main route of HPV infection results from sexual contact, non-sexual transmission (vertical transmission) can occur from mother to child pre- or perinatally. Cesarean births may eliminate vertical transmission, but the surgical risks may outweigh potential benefits [88]. Papanicolaou (Pap) tests are used to screen the cervix for cellular abnormalities that may be indicative of pre-cancerous or cancerous lesions and these cells can be tested for the presence of HPV DNA through reflex testing by *in vitro* nucleic acid hybridization assays. Reflex testing is performed only if there are abnormal Pap results, at which time HPV testing is requested and detection of high risk or low risk HPV types is possible [89]. Pap tests can detect the early stages of cervical cancer termed cervical intraepithelial neoplasias (CIN). These are pre-malignancies graded on a scale of 1–3, with CIN1 indicating only slight dysplasia while CIN2 and CIN3 indicate moderate to severe disease. Diagnosing CIN lesions prior to progression to cervical cancer drastically improves patient survival and response to treatment [90].

The peak age of infection for women is under age 25, with the potential for continual infections by either the same or different HPV types over time [91]. Continual HPV infection increases the risk of transformation, as the likelihood of genome instability and oncogenic mutations rises when HPV is persistent and disruptions to the cell cycle and many other biological processes occur. However, only a small fraction of women will go on to develop cervical cancer. Immune cells clear these infections in most cases, suggesting immune deficiencies, mutations or inherent genomic instability may be contributors to the development of malignancy. Furthermore, whether viral load and disease severity are positively correlated has been controversial in the literature, and may be dependent on the HPV type; however, viral presence and disease risk can now be reliably assessed by E6 and E7 mRNA expression and may be utilized in conjunction with clinical data to more accurately answer this question [7,90].

#### 3.1.2. Head and Neck Cancer

Head and neck squamous cell carcinomas (HNSCCs) account for approximately 50,000 new cancer diagnoses in the United States each year [92]. These tumors are derived from the epithelial cells that line the mucosal surfaces of the head and neck, which include the oral cavity and oropharynx. Previously, common risk factors were tobacco and alcohol use, but more recently HPV infection has emerged as an additional etiological factor in this disease, particularly in oropharyngeal cancers [9]. In fact, the number of oropharyngeal cancers is on a steep upwards trajectory, attributable to increased incidence of HPV-associated tumors [93]. Patients with HPV positive tumors have improved survival rates and respond better to therapies than their HPV negative counterparts [94]. This had led to the suggestion that HNSCCs may be treated as distinct entities, dependent on the HPV status of the tumor. Treatments for HNSCCs include chemotherapy, radiation, and surgery, all of which can lead to undesirable side effects for patients such as hearing loss, dysphagia, facial disfigurement, and inability to use their natural voice. The combined efforts of clinicians and basic scientists to improve the survival rate of HNSCC have been relatively unsuccessful overall [95]. Further identification of how HPV positive and HPV negative HNSCCs differ molecularly will be important for identifying specific therapeutic targets in each subset and a better understanding of the pathogenesis of this disease for improved targeted therapies.

Table 1 depicts a summary of clinical characteristics of cervical and head and neck cancers. Almost all HNSCCs that harbor HPV DNA contain HPV16, whereas in cervical cancer, many more high-risk types of HPV are accountable for the disease. It is not understood why there is such HPV-type specific variability between these cancer types, but perhaps there is a tissue specific or viral exposure preference for HPV16 in the oropharynx over other HPV types. HPV-infected head and neck cancers are mostly localized to the oropharynx, which includes tonsillar and base of tongue cancers, with some evidence that oral cavity cancers have a low prevalence of HPV [96,97]. Unlike cervical cancer, there are no available screening tools such as Pap smears to aid in the prevention of HNSCC development. Designing such screens will be difficult due to the feasibility of accessing the appropriate tissue where infection occurs [98]. Instead, patients must rely on diligently evaluating themselves for persistent symptoms such as sore throats, swollen glands or oral lesions. These seemingly benign symptoms are often overlooked, contributing to the unfortunate fact that HNSCCs are oftentimes not diagnosed until the later stages of disease.

**Table 1:** Summary of clinical characteristics of cervical and head and neck cancers.

	Cervical SCC	Cervical Adenocarcinoma	HPV^+^ HNSCC	HPV^−^ HNSCC	References	
HPV Types (decreasing prevalence)	16 (>50%) 18, 45, 31, 33	18 (38%), 16 (12%), 45, 31	16 (90%), 18, few other HR-HPV types	N/A	[9,86,87]	
Prevalence	~73% of all invasive cervical carcinomas Over 99% HPV^+^	~14% of all invasive cervical carcinomas Over 99% HPV^+^	>25% of all HNSCC	≤75% of HNSCC	[9,99]	
Additional Risk Factors	Smoking, HIV infection, Chlamydia, Oral Contraceptives (>5 years)		Tobacco Usage, Alcohol, Paan (Asia), Maté (South America)		[92,100]	
Incidence	Decreasing USA; High Incidence in Developing Countries	Increasing Incidence	Increasing; younger patients	Decreasing; older patients	[90,95]	

### 3.2. Prognostic Comparison and Staging

In general, the prognosis of cervical cancer is excellent at early stages, often with surgical resection alone. Cervical cancer screening in developed countries has led to early detection, thus dramatically reducing cervical cancer mortality. Unfortunately, locally advanced cervical cancer and metastatic disease is much more common in developing countries making it the second leading cause of cancer related death in women worldwide [101]. Women with cervical cancer may present with abnormal or post-coital vaginal bleeding, but most are asymptomatic and therefore, without proper screening detection is delayed. Cervical cancer is the only cancer type that is still staged clinically and criteria are mostly based on local and distant extension of the tumor (Table 2) [102]. Cervical cancer patients with stage I disease have five-year overall survival rates of 80%–99%; however, survival is greatly reduced to only 15%–16% in those with stage IV disease, even with aggressive treatment [103].

HNSCC is a much more heterogeneous group of cancers, and therefore, the prognosis varies with site and stage of disease. Much like cervical cancer, however, many localized HNSCCs (stage I and II) are cured with radiation or surgical resection alone. As in cervical cancer, HNSCC survival decreases with advancing stage. Importantly, the prognosis of oropharyngeal HNSCC has greatly improved over the past several decades (Table 2). In fact, for tonsil cancer, five-year survival rates have improved dramatically from the 1980s to 2006 in local disease, locally advanced and distant disease, from 62% to 85.9%, 38.2% to 73%, and 21.2% to 41.5%, respectively (Table 2). These improvements are likely reflective of more sophisticated surgical and radiation techniques, increasing HPV prevalence, and better supportive care. However, a more detailed understanding of disease mechanisms will help elucidate strategies to continue to improve these outcomes. In this regard, cooperative group studies investigating treatment paradigms for the earlier and intermediate stage cancers are ongoing. Unfortunately, up to 60% of HNSCCs present locally advanced, and more than 50% of HNSCC develop locoregional recurrence or distant metastasis even with initially aggressive treatment. Upon recurrence or metastasis, the prognosis is dismal at only 10%–15% with a median overall survival of 10–12 months [104]. These cancers prove the most challenging, and successful treatment will thus require intense investigation.

**Table 2:** Staging Criteria and Prognosis for Squamous Cell Carcinomas.

Cervical Cancer				HNSCC *		
Stage	Staging Criteria	Treatment	Prognosis (5yr OS)	Staging Criteria	Treatment	Prognosis (5yr OS) *****
**I**	Carcinoma confined to cervix	Surgical resection (stage IB may benefit by R ± C **)	80%–93%	Tumor less than 2 cm, no LN *** involvement	Single modality (radiation or resection); adverse features at resection = adjuvant R ± C	85.9% for local disease
**II**	Extends beyond cervix, but not pelvic wall	Stage IIA: Surgical resection w/adjuvant R ± C; Stage IIB: R ± C	58%–63%	Tumor between 2–4 cm, no LN involvement		
**III**	Carcinoma extends to pelvic wall and/or involves lower third of vagina and/or causes hydronephrosis or non-functioning kidney	Combined chemoradiation	32%–35%	Tumor >4 cm w/o LN involvement; or tumor <4 cm with single ipsilateral LN <3 cm involvement	Chemoradiation; if residual disease, salvage surgery or resection with adjuvant R ± C	73% for regional disease (III, Iva, IVb)
**IV**	IVa: Extends beyond true pelvis or has clinically involved the mucosa of bladder or rectum; IVb: metastatic disease	IVa: Combined chemoradiation IVb: Palliative chemotherapy	15%–16%	Invasion of tumor in surrounding structures with or without LN involvement; or any tumor with contralateral or >6 cm LN; IVc: metastatic disease	IVa: chemoradiation, induction chemotherapy or resection **** ± adjuvant chemoradiation; IVb: clinical trials *vs.* chemoradiation; IVc: palliative chemotherapy	41.5% distant IVc

*: oropharynx, other sites will vary slightly; **: R ± C: radiation ± chemotherapy; ***: LN: Lymph node; ****: + ipsilateral or bilateral neck dissection based on LN involvement required; *****: Tonsillar Cancer from 2002–2006 [105].

Cervical cancers, whether SCC or adenocarcinoma, have a very high prevalence of HPV. Therefore, it is difficult to determine whether HPV infection per se affects prognosis in these populations. Recently published data suggests HPV is a prognostic factor in anal cancer, predicting both overall and disease-specific survival [106]. However, there have been reports that cervical cancers positive for HPV18 carry a poor prognosis when compared to those positive for HPV16 [107]. In contrast, HPV is associated with fewer HNSCC cases, only around 25%, and almost exclusively with the HPV16 subtype and predominantly in the oropharynx. Substantial evidence has recently emerged demonstrating greater survival for HPV positive HNSCC patients; however this protective effect is lessened in those who report tobacco and alcohol use [94,108].

### 3.3. Treatment Variations

In general, early stage cervical cancer (Stage IA or IB) is treated with resection alone either by conization for those with early stromal invasion or extrafascial hysterectomy for those with lymphovascular invasion. Patients who desire pregnancy may also be candidates for radical trachelectomy with pelvic lymphadenectomy. Modified radical hysterectomy is often offered for patients with stage IA2 or microscopic IB disease. However, stage IB-IIA with high risk pathologic features (positive surgical margin, parametrial invasion, ≥3 positive pelvic lymph nodes or poorly differentiated tumor) requires radical hysterectomy and adjuvant concurrent radiation and chemotherapy (CRT). Adjuvant radiation is recommended for patients with intermediate risk factors (≥50% stromal invasion, lymph-vascular invasion, and tumor size ≥4 cm). Radiation therapy alone is also an acceptable option for early stage disease.

In contrast, locally advanced cervical cancer (Stage IIB–IVA), is treated with radiation alone or CRT. Combined treatment has greatly increased survival in locally advanced disease. The addition of cisplatin treatment was shown to increase overall survival (OS) in several studies including Radiation Therapy Oncology Group (RTOG) 90-01 demonstrating 67% OS in chemotherapy arm *versus* 41% in radiation alone arm at 8 years, Gynecologic Oncology Group (GOG)-120 with 65% in chemotherapy *versus* 47% in radiation alone, and GOG-123 with 83% OS for chemotherapy *versus* 74% with radiation alone [109,110,111].

Patients that develop recurrent disease often undergo either radiation or surgery depending on previous treatments, although some cases may not be amenable to either option. Therefore, these patients and those with metastatic disease are considered incurable and many opt for palliative chemotherapy. In general, chemotherapy is cisplatin-based but many trials have demonstrated increased response rates for combination therapy combining cisplatin and most commonly, paclitaxel. Unfortunately, despite combinations of chemotherapeutics response rates remain low at 25%–35% in recurrent and metastatic disease highlighting a dire need for novel agents [112,113].

Like cervical cancer, HNSCC is often curable with surgical resection or radiation therapy alone for those with early stage disease. However, many of these patients will develop recurrence and oftentimes present with locally advanced disease. Patients with locally advanced disease may undergo surgery, radiation, or CRT with each treatment carrying certain consequences. Surgical resection can result in disfigurement, poor wound healing and at times loss of voice (in the case of laryngeal tumors). However, CRT may result in dysphagia, mucositis, neurotoxicity and renal failure. Despite aggressive treatments, many tumors recur, and patients then undergo the treatment modality not previously offered, or re-resection.

Despite improvements of surgical technique, the rate of relapse for both local and metastatic disease after surgery alone is high, particularly in patients with high-risk features such as perineural invasion, multiple lymph node involvement, extracapsular spread and positive surgical margins. Two pivotal studies demonstrated improved progression-free survival (PFS) and overall survival (OS) in patients with high-risk features treated with adjuvant CRT, albeit with greater toxicity over treatment with radiation alone. However, PFS and OS were only 47% and 53%, respectively at 5 years, suggesting that new treatments are necessary in order to improve outcomes in high-risk patients [114,115].

Combined CRT is frequently offered upfront in locally advanced disease. In 2003, a pivotal phase III trial showed that concurrent chemotherapy and radiation using cisplatin resulted in superior outcomes when compared to radiation alone in unresectable, locally advanced disease. Three year overall survival was 23% in the radiation alone arm compared to 37% in the CRT arm (*p* = 0.014) [116]. As in cervical cancer, cisplatin is the most commonly used chemotherapeutic with the highest response rate. This therapy remains a standard of care for patients with unresectable, locally advanced disease and is also offered for organ preservation. Since the original report, many retrospective studies suggest improved survival in these patients with survival rates as high as 90% at 3 years in some instances [117,118]. Interestingly, the EGFR-directed monoclonal antibody, cetuximab, and concurrent radiation also resulted in an increase in survival in HNSCC of 45.6% *versus* 36.4% for radiation alone; thus, cetuximab is an acceptable agent oftentimes associated with less severe toxicities [119]. Most of the patients in the latter study had oropharyngeal HNSCC, but unfortunately HPV status was unknown. Therefore, it is unclear whether improved survival may have been due to a higher proportion of HPV positive tumors.

Many HNSCC relapses occur locally despite successful initial definitive treatment. Upon local relapse, standard of care is often salvage surgical resection which confers local control rates of 33%–50% and a long term overall survival of 20%–40% [120,121,122]. Many patients, however, are not candidates for resection due to multiple comorbidities. Like cervical cancer, palliative chemotherapy, either single or multiple agent, is regularly offered to these patients as well as to those with metastatic disease. Unfortunately, response rates are poor at 15%–36% with increased toxicity and overall survival rates of only 5–11 months [123,124,125,126].

Our ability to determine which patients (cervical cancer and HNSCC) will recur and which will do well with less intensive therapy and therefore less treatment related toxicity is limited. Active research is ongoing to develop model systems that resemble human HNSCC and that may be useful platforms for the development of alternative treatment strategies [127,128]. As mentioned above, it is unclear whether HPV has an influence on cervical cancer survival as almost all of these tumors are HPV positive, but certainly HPV status has an established impact on the prognosis of HNSCC. In fact, a new report suggests that HPV is not only protective against initial disease progression, but also improves survival in patients with recurrent disease [129]. Patients with HPV positive disease may be able to undergo less intensive therapy, thereby decreasing the incidence of toxicity, at least partly due to their increased sensitivity to radiotherapy [130,131]. Several clinical trials are ongoing including Radiation Therapy Oncology Group (RTOG) Trial 1016 comparing cetuximab and radiation *versus* cisplatin and radiation in locally advanced disease. The benefit of cetuximab in cervical cancer is unknown and actively investigated. However, it is clear that not all HPV positive HNSCC tumors behave equally. Positive smoking history, likely resulting in increased genomic mutations, may explain why some HPV positive tumors do not fare as well with therapy. Accordingly, the improved survival of HPV positive patients is negated by tobacco use [108]. As the incidence of this group of cancers grows, it is important to further classify these tumors and elucidate optimal targets that are specific to each subset.

### 3.4. Vaccines Offer Protection from HPV-Associated Cancers

Cancers often arise due to mutations that are either inherited, occur *de novo*, or are acquired from exposure to environmental factors. As cancers are quite heterogeneous in nature, with a multitude of mutations, identifying therapies that work on entire tumor cell populations including tumor-initiating or cancer stem cells has been difficult [132]. Current therapeutic intervention includes radiation, chemotherapy, and surgery, with the major caveat that many of these treatments cause major disruptions in a patient’s quality of life. The transforming ability of human papillomavirus in the cervix, anogenital tract, and oropharynx suggests that prevention of HPV infection may ultimately prevent HPV-associated cervical and head and neck cancers. Vaccination has been successful at preventing infection of other viruses including influenza, rotavirus, and varicella, setting the precedent and opportunity for scientists to create a vaccine that prevents infection with HPV and therefore, HPV-associated cancers.

There are currently two vaccines on the market: *Cervarix*^®^ (GlaxoSmithKline, Brentford, Middlesex, UK) and *Gardasil*^®^ (Merck & Co., Whitehouse Station, NJ, USA). These vaccines cover the most common oncogenic HPV types known to cause cervical cancer, while *Gardasil*^®^ also includes those responsible for genital warts. *Cervarix*^®^ is a bivalent vaccine that protects against high risk HPV16 and 18, while *Gardasil*^®^ is quadrivalent and protects against HPV16 and 18, as well as the low risk HPV6 and 11 types. Additionally, protection for both vaccines has been demonstrated to be ≥5 years [133,134,135]. These vaccines are protective against cervical, vaginal and vulvar cancers, as well as genital warts in the case of *Gardasil*^®^. The current recommendation by the Food and Drug Administration (FDA) is for men and women ages 9–26 years to be vaccinated. This is the age range where HPV infection is most likely to occur. It is imperative to begin the vaccination series at the earliest recommended ages, prior to the start of sexual activity, to offer maximal protection against HPV infection; vaccination after infection does not result in enhanced protection from or clearance of the virus.

Both *Cervarix*^®^ and *Gardasil*^®^ utilize virus-like particles (VLP) formed by self-assembly of the HPV capsid protein L1 to elicit the immune response, as opposed to live or attenuated virus. Antibodies are then produced against the L1-specific to each HPV type included in the vaccine [136]. Although capsid proteins are highly homologous amongst HPV types, it is unclear how much protection is offered against other HPV types not covered by vaccination; however, a recent study with *Cervarix*^®^ suggests it may also cross-protect against the oncogenic HPV types 31, 33, 45 and 51 [137]. Since the introduction of these vaccines in the mid-2000s, wide spread studies of their efficacy in various populations and countries has been performed. These studies have focused on anogenital cancers and genital warts, but the vaccines are not yet approved as preventives against HPV-infected HNCs, partially impeded by the lack of information on HNCs required by the FDA for future studies [138,139]. Unlike cervical cancer where there is still a large prevalence of other high-risk HPV types contributing to disease, over 90% of all head and neck cancers are HPV16 positive. Even though not yet approved by the FDA for protection against HNCs, these vaccines may be highly efficacious as they cover the HPV type that is overwhelmingly implicated in HNC carcinogenesis.

## 4. Molecular Comparisons between Cervical and Head and Neck Cancers

### 4.1. Mutational Analyses

The advent of whole exome and whole genome sequencing and other “omics” technologies has enabled scientists to identify specific mutations in tumor specimens that may lead to targeted and personalized therapies. This is especially important in cancers such as cervical and head and neck where a diagnosis is often not made until the advanced stages of disease. These late stage tumors are not as treatable and have few targetable molecules identified. Previously, common chromosomal aberrations in both tumor types had been identified in the presence of HPV. These include loss at 13q and 11q22.3–25 and gains at 20q and 3q24–29; albeit unique chromosomal alterations also exist for cervical and HNC [140]. This indicates the existence of specific genomic alterations which are caused by the presence of high risk HPV independent of anatomical site. Only recently have samples from these two cancer types been analyzed by whole exome sequencing, which revealed profiles of somatic mutations and globally de-regulated pathways.

A recent publication by Ojesina *et al*. [141], compared mutations in cervical squamous cell carcinoma and adenocarcinoma against normal controls to identify novel mutations. In this study the authors identified multiple new mutations in cervical cancer including *MAPK1*, *FBXW7*, and *EP300* [141]. Additionally, pathway analysis revealed that genes associated with the immune response were most significantly mutated in cervical SCC, while cervical adenocarcinoma had recurrent mutations in the PIK3CA/PTEN pathway.

Similar publications from exome sequencing of HNSCCs were reported in 2011. Only a few somatic mutations match those discovered in the cervical cancers [142,143], including *FBXW7* and *PIK3CA*. The head and neck studies were further segmented into HPV^+^ and HPV^−^ categories, with far fewer genes mutated in HPV^+^ as compared to HPV^−^ tumors. Not surprisingly, HPV^−^ tumors harbored higher global mutation frequencies compared to the HPV^+^ group, presumably due to tobacco use and highlighted by *TP53* mutations in almost all HPV^−^ tumors. Table 3 depicts prominent examples of mutated genes in both the cervical and head and neck cancer studies. A complete list can be obtained from the original research papers. The data suggest that although HPV can be the etiological cause in both cancer types, associated cellular mutations are variable. This may be due to other etiological influences that drive mutations including tobacco use or to the differential anatomical locations of the tumors.

**Table 3:** Mutations identified by whole exome sequencing. Mutated genes that are shared between cancer types.

Cervical SCC [141]	HPV^+^ HNSCC [142,143]	
FBXW7 PIK3CA MAPK1 HLA-B STK11 EP300 NFE2L2	NOTCH1 SYNE1 HRAS PIK3CA MED1 MLL2 EZH2 SYNE2 NOTCH3 [142]	FBXW7 CDKN2A NOTCH1 PIK3CA [143]

### 4.2. HPV Integration

HPV integration in cervical cancer can lead to the disruption of E2 as HPV enters common fragile or other sites in the host genome. This is an important event during the progression of cells to a transformed state, albeit not necessarily required, which provides a growth advantage given the control of E6/E7 expression by E2 under normal circumstances. Transformation is continuously dependent upon E6/E7 expression and can be reversed by the reintroduction of E2 [144,145,146]. Research related to the mechanisms and sites of HPV integration has been limited to cervical cancers, with nascent studies of integration in HNCs. Integration is one of the more poorly understood aspects of HPV biology, with controversy over where integration events occur: either randomly through the genome or at specific “hotspots”. These “hotspots” include cytogenetic bands 3q28, 4q13.3, 8q24.21, 13q22.1, and 17q21.2 accounting for integration sites of 22% of cervical tumors analyzed [147,148]. This study also included strong evidence for HPV DNA near various miRNA sites, some of which are already published as known regulators in cervical cancer. On the other hand, recent work demonstrates HPV integrates randomly across the genome, but tends to cluster near sites of structural alterations in the genome [149]. This report included not only cervical cancer lines, but also HNC cell lines and primary tumor specimens, all exhibiting a similar pattern of HPV integration in regions of genomic instability. An elegant mechanism to explain this phenomenon was proposed, with a viral genome looping model to explain HPV-driven amplifications and rearrangements that occur at sites of integration, which may be continually propagated throughout the genome [149,150]. Additionally, the authors identified numerous breakpoints in the integrated HPV genome that are outside of the E2 open reading frame, thus questioning the longstanding model of preferential E2 disruption through integration.

Even with some disparate findings between the above reports, both identify *TP63* as a gene affected by integration events found in both cervical and HNCs. Schmitz *et al*. defined the chromosomal hotspot 3q28, where the *TP63* gene is located, and demonstrated *TP63* was disrupted by HPV integration in multiple cervical tumors. Likewise, Akagi *et al*. identified HPV integration within the *TP63* gene, resulting in the loss of normal *TP63* gene expression and the creation of a novel protein isoform, along with viral-host fusion transcripts. Furthermore, c-myc was also amplified and overexpressed in both cancer types in the presence of integrated HPV [151,152]. Although E6 and E7 are sufficient for immortalization *in vitro*, the accumulation of oncogenic mutations in cellular tumor drivers or suppressors is necessary to achieve neoplastic transformation and cancer development [46]. In depth studies enabled by next-generation genomics and functional studies will dissect the contribution of HPV integration and genomic rearrangements to carcinogenesis. Elucidating the role of the full complement of human genes whose activation or inactivation initiate and drive transformation will identify new targets and therapies tailored to the mutational landscape of individual tumors and patients.

### 4.3. Gene Expression in HPV^+^ Cancers

It is clear that HPV infection and transformation de-regulates gene expression patterns in keratinocytes. For many years, studies of HPV-driven host gene expression involved either the introduction of the HPV genome or of the HPV E6 and E7 oncogenes in existing cell lines. More recently, studies of primary tumors compared to control specimens have provided additional insights in clinically relevant model systems. To create a database of reliable genomics and transcriptomics information for many cancer types, the National Cancer Institute (NCI) along with the National Human Genome Research Institute (NHGRI) collaborated on the ambitious “The Cancer Genome Atlas” (TCGA) project [153]. The goal of this project was to create freely available tumor datasets (with appropriately matched normal controls) subjected to genomic studies including exome sequencing, single nucleotide polymorphism (SNP) analysis, and mRNA expression by RNA-Sequencing, together with extensive clinical annotations. Currently, information from over 30 tumor types has been deposited in the TCGA, with a collection target of at least 500 samples for each cancer type [153]. The TCGA will provide massive datasets for broad integration with basic and clinical sciences. This includes the opportunity for personalized medicine, where new diagnostics, therapeutic targets, and individualized treatments may eventually be possible. Both cervical squamous cell carcinoma and adenocarcinoma, along with HNSCC are part of the TCGA; however, the cervical data will not be available for data mining until later in 2014. Once these data are available, it will allow for groundbreaking opportunities to compare HPV^+^ cervical with HPV^+^ HNCs. Given the large number of specimens already banked for both cancer types, clear trends in gene expression should delineate common molecular and genomic occurrences that are due to HPV activities.

In the absence of published TCGA based comparisons, smaller studies that profile HPV-related gene expression are publically available. One of the first papers published comparing cervical cancer (SCC and adenocarcinoma) to normal cervical keratinocytes identified approximately 500 genes up- or down-regulated in the presence of HPV [154]. Up-regulated genes included *CDKN2A* (p16), *TOP2A*, *E2F1*, *FOXM1*, *MCM2/4/5*, and *PTGES*, with a high prevalence of genes involved in cell cycle regulation. Down-regulated genes including *TGFβ1*, *TGFα*, and *KRT16*, were clustered in differentiation and tumor suppressor pathways [154]. This report also demonstrated substantial similarities in expression profiles of cervical SCC and adenocarcinomas, despite the fact that SCCs were HPV-16 positive and adenocarcinomas were HPV-18 positive. This suggested distinct high-risk HPV types evoke similar transcriptome alterations [154].

Analyses in 2013 utilized a retrospective approach to identify novel genes involved in dysplastic and neoplastic cervical lesions, based on targets previously published in the literature [155]. The authors confirmed earlier results where *FOXM1*, *TOP2A*, and *E2F1* were not only overexpressed, but where their respective expression levels gradually increased as cervical lesions progressed from low-grade to high-grade lesions and eventually to carcinomas. Other de-regulated genes included *CDC25A*, *TP73*, and *CCNE2*. Conversely, genes that demonstrated reduced expression in the progression from dysplastic tissue to carcinoma included *PTGS2*, *BCL2*, and *FOS* [155].

A multitude of publications comparing the gene expression profiles of HPV^+^ to HPV^−^ HNSCCs are available. One study compared HPV^+^ *versus* HPV^−^ *versus* normal oral mucosa and analyzed these specimens in a number of ways [156]. The authors identified many of the same gene expression changes reported previously including the up-regulation of *CDKN2A*, *TOP2A*, and *MCM2/3* in HPV-infected HNCs compared to normal mucosa. Other up-regulated genes reflected pathways such as DNA replication, cell cycle progression, DNA repair and differentiation, while down-regulated genes reflected processes such as proteolysis and chemotaxis. Additional comparisons between HPV^+^ and HPV^−^ HNCs identified up-regulated genes involved in nuclear structure, mitosis, DNA repair and methylation, and down-regulated genes involved in signal transduction and proteolysis [156]. Finally, another report directly compared gene expression of HPV^+^ cervical and head and neck cancers and discovered similar trends where cell cycle-related genes are highly up-regulated [157]. Other datasets confirm these findings where HPV drastically affects gene expression patterns involved in cell cycle, repair and replication [157,158,159,160]. This is not necessarily surprising given the known function of E6 and E7 in the inhibition and degradation of critical tumor suppressors such as p53 and Rb.

p53 and Rb-dependent functions are regulated by p14ARF and p16, respectively, which are encoded by the same gene, *CDKN2A* [161,162]. This is one of the most frequently overexpressed genes in HPV positive tumors, largely due to regulatory interference by E6 and E7. When E7 inactivates Rb, E2F1 is free to initiate transcription of its targets that includes the *CDKN2A* locus [163]. p16 is so consistently and highly overexpressed in HPV^+^ cancers that it is used clinically as a surrogate marker for HPV positivity during cancer diagnosis.

Epigenetics is another mechanism whereby gene expression patterns can be altered in normal and transformed cells. This is a complex field that investigates DNA changes that do not affect the inherent nucleotide sequence, but rather processes such as methylation patterns and histone modifications. The presence of HPV greatly affects methylation signatures across the genome, promoting hyper- and hypomethylation. For example, tumor suppressor genes, such as TERT, exhibit promoter hypermethlyation and thus gene silencing in CIN lesions and cervical cancers [164,165]. The methyltransferases responsible for methylation, DNMT1, DNMT3A, and DNMT3B, are also highly overexpressed in HPV positive cervical and HNSCC cells [90,166,167]. Conversely, hypomethylation increases during the transition of dysplastic to cancer cells and results in aberrant gene activation, suggesting that hyper- and hypomethylation coordinate for the evolution of an oncogenic state [168]. Comparisons of HPV positive and negative HNSCC samples also reveal the extent to which HPV alters methylation patterns, research which contributes substantially to the identification of novel target genes [169].

Initial studies comparing cervical and HNCs to normal controls suggested HPV elicits similar gene expression alterations between tumor types. Publication and analysis of the TCGA data will likely confirm this trend, but novel details are also expected which should reflect differential tumor origins in various tumor types, data which may be exploited for the targeting of tumor cells by novel agents. The large number of samples combined with thorough bioinformatics analyses and ultimately confirmatory studies will help validate genes of interest shared or unique to cervical and HNCs. Further separation of these groups based on clinical, environmental and demographic data is bound to enrich and surprise the HPV community.

## 5. Conclusions

Human papillomavirus is the most common sexually transmitted viral pathogen and causes approximately 5% of the global cancer burden [170]. Even after decades of intense research of the epidemiology and molecular biology of HPV infection and transformation, the aggressive nature of HPV-related late stage cancers and novel molecular targets for new therapies do not meet clinical needs. Cellular events downstream of HPV infection and integration such as the induction of genomic instability and selection for oncogenic mutations must occur in order to complete transformation process that is initiated by the expression of the E6 and E7 oncogenes. Cervical cancer was the first tumor type attributed to the effects of HPV infection and has since been the standard for studying HPV biology. In fact, one of the most widely used cell lines in cancer biology is the HeLa cell line derived from an HPV18 cervical adenocarcinoma. The discovery that HPV infection is also an underlying cause of head and neck squamous cell carcinomas has opened the door for comparisons that will ultimately inform the treatment of each tumor type towards improved diagnostics, therapeutic options, and patient survival.
